# Sexual dimorphism in pelvic measurements of Wistar rats (*Rattus norvegicus*) during pre- and post-pubertal periods: a 3D morphometric study

**DOI:** 10.1186/s12917-026-05743-x

**Published:** 2026-07-18

**Authors:** Remzi Orkun Akgun, Zuhal Ovuz, Yunus Emre Atay, Ufuk Kaya

**Affiliations:** 1 Department of Basic Sciences, Faculty of Dentistry, Cankiri Karatekin University, Cankiri, TR18100 Türkiye; 2 Department of Maxillofacial Radiology, Faculty of Dentistry, Cankiri Karatekin University, Cankiri, TR18100 Türkiye; 3 Department of Obstetrics and Gynecology, Faculty of Veterinary Medicine, Erciyes University, Kayseri, TR38280 Türkiye; 4 Department of Biostatistics, Faculty of Veterinary Medicine, Hatay Mustafa Kemal University, Hatay, TR31060 Türkiye

**Keywords:** Wistar rat, Pelvic morphometry, Sexual dimorphism, Puberty, Three-dimensional imaging

## Abstract

**Background:**

Sexual dimorphism and developmental stage are key determinants of skeletal morphology in mammals. The pelvic complex, which plays an essential role in locomotion and reproduction, undergoes significant morphological changes during growth and maturation. Advances in three-dimensional (3D) imaging technologies have enabled highly accurate morphometric analyses of skeletal structures. We hypothesized that (i) pubertal development would significantly affect pelvic morphometric parameters, (ii) males and females would exhibit significant differences in selected pelvic measurements, and (iii) sexual dimorphism would become more evident after the onset of puberty. Therefore, the aim of this study was to evaluate pelvic morphometric parameters in male and female Wistar rats during prepubertal and postpubertal stages using 3D digital models.

**Methods:**

A total of 28 Wistar rats (14 females and 14 males) were included and divided into prepubertal (30–35 days) and postpubertal (65–70 days) groups. Cone Beam Computed Tomography (CBCT) imaging of the pelvic region was performed, and cross-sectional images were exported in DICOM format. 3D pelvic models were reconstructed using 3D Slicer software following bone segmentation. Twelve morphometric parameters (M1–M12) were measured digitally on the reconstructed models by two independent investigators at three different time points. The effects of gender and pubertal status were analyzed using two-way analysis of variance (ANOVA) with Bonferroni-adjusted post-hoc tests.

**Results:**

Pubertal status significantly influenced all evaluated parameters. Highly significant differences were observed for M1–M10 (*p* < 0.001), while M11 and M12 also demonstrated significant pubertal effects (*p* < 0.05). Postpubertal rats exhibited consistently greater measurements than prepubertal animals. Significant gender-related differences were identified in several parameters, and significant gender × puberty interaction effects were detected for selected measurements, indicating sex-dependent growth patterns during maturation.

**Conclusions:**

Pubertal maturation represents the principal determinant of pelvic morphometric variation in Wistar rats, while gender contributes additional structural differences. These findings may provide baseline morphometric data and suggest that both developmental stage and sex should be considered when interpreting morphometric outcomes in experimental and comparative anatomical research.

## Background

 Rodents are the largest group of mammals, found on every continent except Antarctica. Globally, there are 2552 rodent species, among which three-the house mouse (*Mus musculus*), the brown rat (*Rattus norvegicus*), and the black rat (*Rattus rattus*)-inhabit a wide range of environments and occur at higher population densities than any other rodent species [1]. The laboratory rat (*R. norvegicus*) belongs to the order *Rodentia* and the family *Muridae*. The genus *Rattus* includes over 130 species, but *R. norvegicus* and *R. rattus* are the two most commonly encountered. Laboratory rats are second only to mice as the most frequently used mammals in biomedical and behavioral research [2]. Moreover, Wistar rats and Sprague-Dawley rats are increasingly becoming the most widely used laboratory animals worldwide [3].

Sexual dimorphism is defined as the morphological differences in size or shape of specific anatomical structures between the sexes of a species. In mammals, although certain sexually dimorphic traits may be present at birth, these differences typically become more pronounced during ontogenetic development. The pelvic complex in mammals exhibits sexual dimorphism in both its dimensional proportions and morphological configuration. In mammals, the coxa bone (*os coxa*) is formed bilaterally by the fusion of three endochondral bones: the ilium (*os ilium*), the ischium (*os ischium*), and the pubis (*os pubis*). The right and left *os coxa* articulate with the sacral bone (*os sacrum*) to form the pelvis. This complex structure exhibits sexual dimorphism in both shape and size across nearly all mammalian species, including those without pronounced differences in gross body size or shape [4].

Morphometric differences in pelvic dimensions have been reported in several mammalian species. Comparative studies have demonstrated that pelvic morphology varies substantially among mammals in relation to reproductive demands, locomotor behavior, and developmental factors. For example, pelvic sexual dimorphism has been documented in a wide range of mammalian taxa, with females generally exhibiting relatively larger birth-related pelvic dimensions than males [5]. In rodents, significant sex- and reproduction-related differences in pelvic size and shape have been reported in the bank vole (*Myodes glareolus*), indicating that both sex and reproductive status may influence pelvic morphology [6]. Similarly, studies in non-human primates have revealed species-specific variation in pelvic morphology associated with locomotor adaptations and evolutionary history [7]. Furthermore, geometric morphometric analyses have demonstrated substantial variation in pelvic shape and sexually dimorphic traits within mammalian populations, highlighting the complex interplay of genetic, developmental, functional, and reproductive factors that shape pelvic morphology [8, 9].

Puberty represents a critical developmental transition characterized by accelerated skeletal growth, hormonal maturation, and substantial remodeling of the musculoskeletal system [10, 11]. In rodents, this period is associated with increased secretion of sex steroids that influence bone growth, pelvic development, and the emergence of sexually dimorphic skeletal characteristics [12–14]. Pelvic sexual dimorphism is generally considered a consequence of reproductive differences between males and females. Since newborns must pass through the birth canal formed by the pelvic bones, morphological differences in the female pelvis emerge as an adaptation to childbirth [4]. Nonetheless, pubertal development induces morphological and dimensional modifications in the pelvis, presumably driven by changes in hormonal metabolism [12, 15].

Significant advancements in technology have facilitated the development of three-dimensional (3D) anatomical modeling. A 3D digital model of an anatomical structure can be generated by processing cross-sectional images obtained through Computed Tomography (CT) and Magnetic Resonance Imaging (MRI) using specialized software programs. These imaging techniques produce two-dimensional (2D) cross-sectional images that are stored in a digital format known as DICOM (Digital Imaging and Communications in Medicine). Using software specifically developed for DICOM files, 3D digital models of various anatomical structures can be constructed [16, 17].

In morphometric studies, alongside traditional methods, 3D digital measurements have become a commonly used approach among researchers. Linear and angular 3D digital measurements have been compared with those obtained using digital calipers and goniometers, and their reliability in terms of accuracy has been established [18–20]. It has been stated that 3D digital measurements can be reliably utilized in the analysis of skeletal structures [18, 21]. Numerous morphometric studies utilizing 3D digital modeling techniques on laboratory animals have been reported in the literature. These studies have conducted measurements on various anatomical structures including the skull [21], pelvis [22, 23], antebrachium [24], paranasal sinuses [25], mandible [26], forelimb joints [27], and long bones [28] in laboratory rabbits; long bones [29] and skull [30] in guinea pigs; and airways and pulmonary vessels in rats [31].

Despite the widespread use of Wistar rats in biomedical research, information regarding pelvic morphometric changes occurring during pubertal development remains limited. Previous studies have primarily focused on adult animals, strain comparisons, or two-dimensional radiographic assessments, while the combined effects of sex and pubertal maturation on pelvic morphology have received little attention. Furthermore, 3D morphometric analyses investigating sexual dimorphism during developmental stages in Wistar rats are scarce.

Based on previous evidence indicating that pelvic morphology is influenced by both sex-specific biological factors and developmental maturation, we hypothesized that (i) pubertal development would significantly affect pelvic morphometric parameters, (ii) males and females would exhibit significant differences in selected pelvic measurements, and (iii) sexual dimorphism would become more evident after the onset of puberty. Therefore, the aim of this study was to evaluate pelvic morphometric parameters in male and female Wistar rats during prepubertal and postpubertal stages using 3D digital models. The selected morphometric parameters represent key anatomical dimensions associated with pelvic growth, skeletal maturation, and biomechanical organization of the pelvic complex. Characterization of these parameters may provide baseline reference data for developmental biology, comparative anatomy, and translational studies utilizing rodent models. Furthermore, identifying differences in pelvic morphology during maturation may contribute to a better understanding of skeletal growth patterns and developmental adaptations relevant to orthopedic, reproductive, and growth-related research. By combining 3D digital reconstruction with morphometric analysis, this study also aims to establish reference values that may facilitate future investigations of pelvic development and sexual dimorphism in experimental animal models.

## Materials and methods

### Animals and experimental design

A total of 28 adult Wistar rats (14 females and 14 males) were used in the study. The mean body weights of the groups were 119 ± 3.9 g for prepubertal males, 104 ± 3.1 g for prepubertal females, 310 ± 8.2 g for postpubertal males, and 207 ± 5.6 g for postpubertal females. Throughout the study, rats were housed under standardized environmental conditions (22 ± 2 °C, 50–60% relative humidity, and a 12 h light/12 h dark cycle) and were provided with a standard commercial laboratory rodent diet and water ad libitum. All animals were obtained from the Experimental Animal Unit of Erciyes University and maintained under identical husbandry conditions to minimize environmental and nutritional variability. Prior to specimen collection, all animals had been euthanized using an overdose of anesthetic agents and showed no evidence of skeletal abnormalities, congenital malformations, or pathological conditions affecting the pelvic bones. Male and female rats were grouped into prepubertal (30–35 days old) and postpubertal (65–70 days old) categories based on puberty periods reported in the relevant literature [2, 3], with 7 animals included in each group. All animals were obtained from the Experimental Animal Unit of Erciyes University with appropriate ethical approval. This study was approved by Erciyes University Animal Experiments Local Ethics Committee (Decision no: 24/220).

**Fig. 1:**
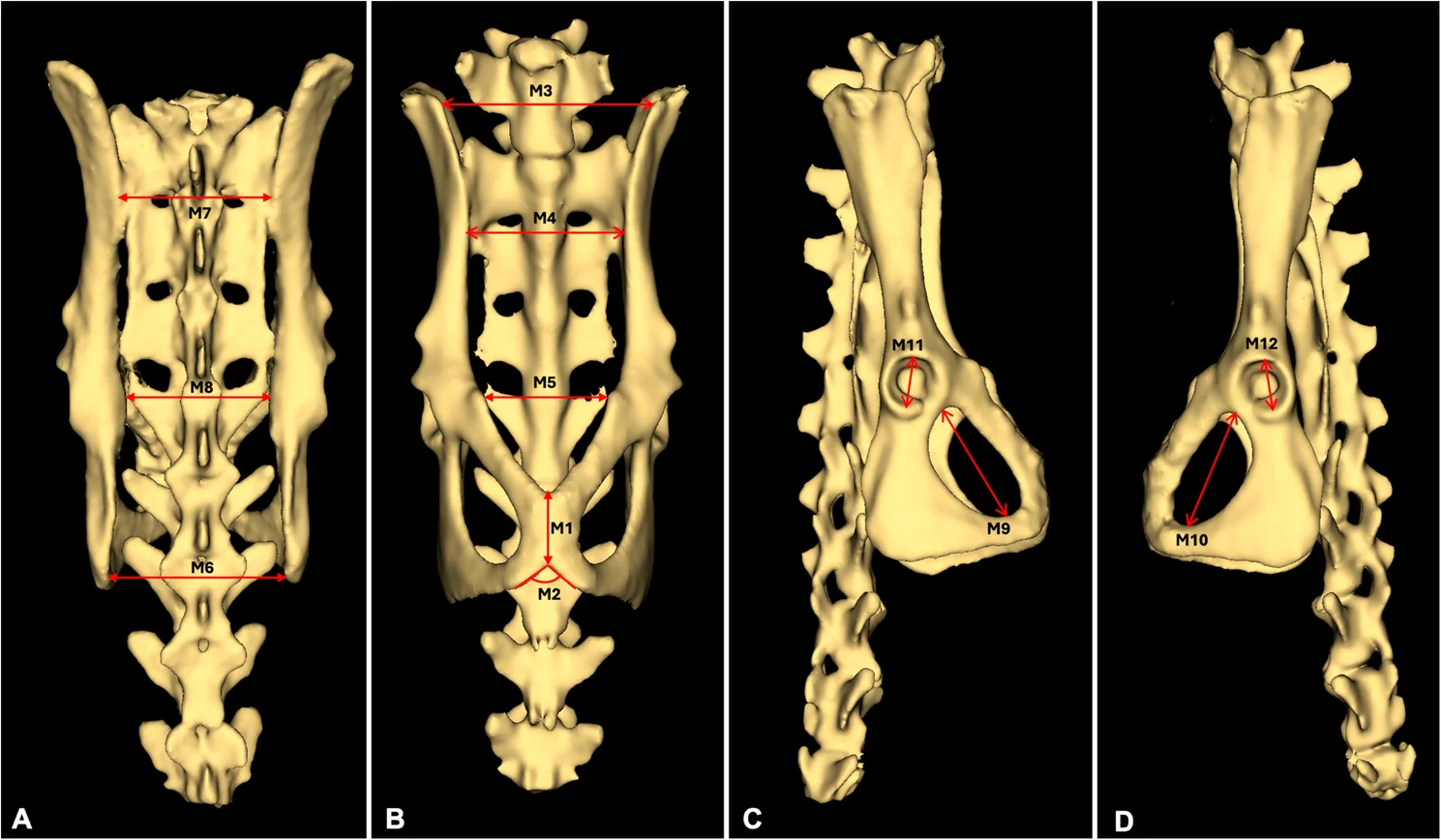
Measurement regions (M1–M12) on the 3D digital pelvic model of a postpubertal male Wistar rat. **A** Dorsal view; **B** Ventral view; **C** Right lateral view; **D** Left lateral view

Cone Beam Computed Tomography (CBCT) imaging of the pelvic regions of the rats was performed. The imaging parameters were set as follows: 10 × 8 cm field of view (FOV), 68 μm voxel size, 90 kV, 4 mA, and 32 mAs. Cross-sectional images were exported in DICOM file format and imported into the 3D Slicer software (Slicer 5.8.1, GitHub, San Francisco) for bone segmentation. Bone segmentation was performed using a threshold-based approach. The threshold range was set between 271.40 and 2031.80 Hounsfield unit-equivalent values to isolate mineralized bone tissue from surrounding structures. Following segmentation, a smoothing procedure was applied to reduce surface irregularities and improve model quality, using a smoothing factor of 0.50. The region of interest (ROI) for pelvic segmentation was defined from the last lumbar vertebra cranially to the fourth caudal vertebra caudally. All skeletal structures within these anatomical limits were included in the segmentation. Following segmentation, the pelvic skeleton was reconstructed into a 3D model. Digital measurements were performed on the 3D models by two independent investigators at three separate time points to ensure methodological rigor and reproducibility. The measurement locations are detailed in Table 1 and illustrated on the 3D pelvic models (Fig. 1). These measurements were selected because they represent major anatomical dimensions of the pelvic inlet, pelvic cavity, and pelvic outlet, and are commonly used in morphometric studies investigating pelvic development, sexual dimorphism, and skeletal growth [4, 22, 23, 32, 33]. Collectively, these parameters allow comprehensive assessment of pelvic size, shape, and developmental changes associated with sex and pubertal maturation. Anatomical terminology and landmark definitions used throughout the study were established in accordance with the Nomina Anatomica Veterinaria [34].

**Table 1:** Definitions of pelvic morphometric parameters (M1–M12)

Measurement (M)	Measurement description	Anatomical landmarks description
M1 (mm)	Length of the pubic symphysis	The linear distance between the cranial and caudal margins of the pubic symphysis measured along its longitudinal axis
M2 (°)	Subpubic angle	The angle formed by the convergence of the right and left ischiopubic rami at the ventral aspect of the pelvic outlet
M3 (mm)	Bi-iliac width	The linear distance between the most lateral points of the right and left iliac Wings
M4 (mm)	Medial transversal diamater	The shortest transverse distance between the medial surfaces of the right and left pelvic walls at the level of the pelvic cavity
M5 (mm)	Bi-iliopubic eminence width	The linear distance between the right and left iliopubic eminences.
M6 (mm)	Bi-ischial width	The linear distance between the most lateral points of the right and left ischial tuberosities
M7 (mm)	Bi-sacroiliac width	The linear distance between the right and left sacroiliac joints measured at their most lateral margins
M8 (mm)	Bi-acetabular width (Inter-medial wall distance)	The linear distance between the medial walls of the right and left acetabula
M9 (mm)	Craniocaudal diameter of the right obturator foramen	The maximum craniocaudal length of the right obturator foramen measured between its cranial and caudal margins
M10 (mm)	Craniocaudal diameter of the left obturator foramen	The maximum craniocaudal length of the left obturator foramen measured between its cranial and caudal margins
M11 (mm)	Craniocaudal diameter of the right acetabulum	The maximum craniocaudal diameter of the right acetabular cavity measured between its cranial and caudal borders
M12 (mm)	Craniocaudal diameter of the left acetabulum	The maximum craniocaudal diameter of the left acetabular cavity measured between its cranial and caudal borders

### Statistical analysis

G*Power software version 3.1.9 was used for the sample size calculation. It resulted that the minimum number of rats was 28, considering an effect size of 0.56, an alpha value of 0.05, and a power of 0.80. Before proceeding to significance testing, the variables were examined for normality assumption using the Shapiro-Wilk test and for homogeneity of variances using Levene’s test. Differences in pelvic measurements regarding gender and puberty factors were determined using two-way analysis of variance (ANOVA). For measurements where statistical significance was determined, Bonferroni-adjusted simple effect analysis was used as a post-hoc test. All statistical analyses were performed using the IBM SPSS 23.0 software package. The level of statistical significance was set at *p* < 0.05.

To assess measurement reliability, both intra-observer and inter-observer agreement analyses were performed using the intraclass correlation coefficient (ICC). For intra-observer reliability, the same investigator repeated all measurements at three different time points. Inter-observer reliability was evaluated by comparing measurements obtained independently by two investigators. ICC values greater than 0.90 were obtained for all morphometric parameters, indicating excellent measurement reproducibility and observer agreement.

## Results

**Fig. 2:**
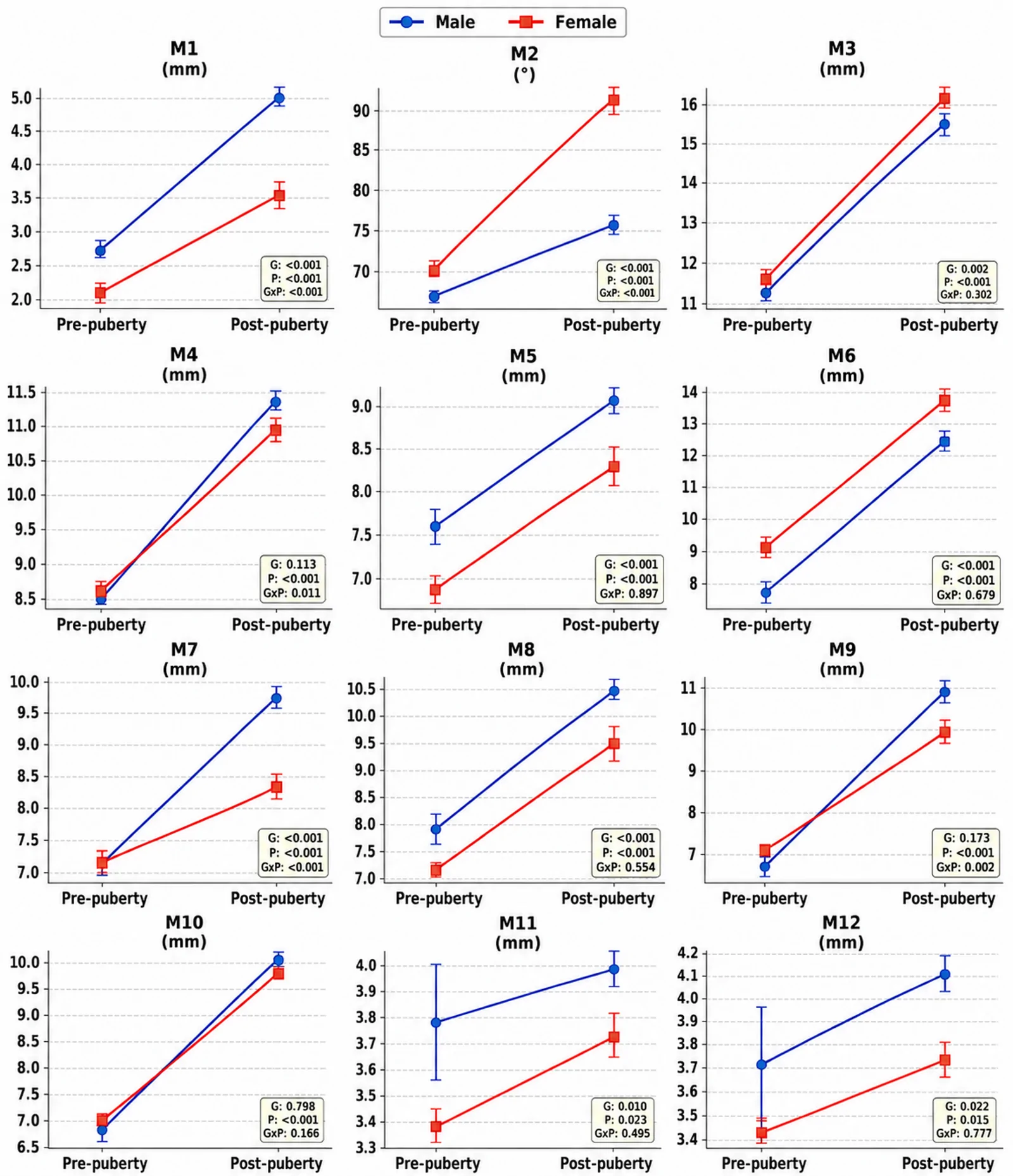
Interaction plots: Gender (G) × Puberty (P) stage effects on pelvic measurements

The effects of gender and pubertal status on the evaluated morphometric parameters (M1–M12) were assessed using a two-way analysis of variance (ANOVA). The analysis revealed that pubertal status exerted a robust and consistent effect across all investigated parameters. All statistical findings are presented in Table 2. To facilitate data interpretation, interaction plots illustrating the effects of gender, pubertal status, and their interaction on all evaluated pelvic morphometric parameters (M1–M12) were generated and are presented in Fig. 2.

**Table 2:** Effects of gender and pubertal status on morphometric parameters (M1-M12) in Wistar rats: mean ± standard error of the mean (SEM) values, least squares (LS) means, and two-way ANOVA results

Puberty										
Parameter	Gender	Pre		Post		LS Mean (Gender)		*P*		
		Mean	SEM	Mean	SEM	Mean	SEM	G	*P*	G x P
M1	Male	2.70 ^b, A^	0.06	5.02 ^a, A^	0.02	3.86	0.32	< 0.001	< 0.001	< 0.001
	Female	2.09 ^b, B^	0.08	3.55 ^a, B^	0.10	2.82	0.21			
LS Mean (Puberty)		2.39	0.10	4.29	0.21					
M2	Male	67.10 ^b, B^	0.32	75.81 ^a, B^	0.82	71.46	1.28	< 0.001	< 0.001	< 0.001
	Female	70.07 ^b, A^	0.20	92.24 ^a, A^	1.12	81.16	3.12			
LS Mean (Puberty)		68.59	0.45	84.03	2.37					
M3	Male	11.28	0.08	15.61	0.20	13.45 ^B^	0.61	0.002	< 0.001	0.302
	Female	11.64	0.14	16.29	0.16	13.97 ^A^	0.65			
LS Mean (Puberty)		11.46 ^b^	0.09	15.95 ^a^	0.16					
M4	Male	8.44	0.03	11.41	0.10	9.93	0.41	0.113	< 0.001	0.011
	Female	8.56	0.12	10.95	0.13	9.76	0.34			
LS Mean (Puberty)		8.50 ^b^	0.06	11.18 ^a^	0.10					
M5	Male	7.57	0.19	9.10	0.14	8.33 ^A^	0.24	< 0.001	< 0.001	0.897
	Female	6.82	0.16	8.31	0.21	7.56 ^B^	0.24			
LS Mean (Puberty)		7.19 ^b^	0.16	8.70 ^a^	0.17					
M6	Male	7.72	0.28	12.39	0.14	10.06 ^B^	0.67	< 0.001	< 0.001	0.679
	Female	9.12	0.27	13.60	0.16	11.36 ^A^	0.64			
LS Mean (Puberty)		8.42 ^b^	0.27	13.00 ^a^	0.20					
M7	Male	7.31 ^b^	0.17	9.76 ^a, A^	0.13	8.53	0.35	< 0.001	< 0.001	< 0.001
	Female	7.33 ^b^	0.15	8.44 ^a, B^	0.13	7.88	0.18			
LS Mean (Puberty)		7.32	0.11	9.10	0.20					
M8	Male	7.87	0.25	10.54	0.15	9.20 ^A^	0.40	< 0.001	< 0.001	0.554
	Female	7.08	0.13	9.55	0.10	8.31 ^B^	0.35			
LS Mean (Puberty)		7.48 ^b^	0.17	10.04 ^a^	0.16					
M9	Male	6.66 ^b^	0.27	10.93 ^a, A^	0.24	8.80	0.62	0.173	< 0.001	0.002
	Female	7.08 ^b^	0.11	9.96 ^a, B^	0.10	8.52	0.41			
LS Mean (Puberty)		6.87	0.15	10.45	0.18					
M10	Male	6.85	0.28	10.13	0.07	8.49	0.48	0.798	< 0.001	0.166
	Female	7.04	0.11	9.86	0.09	8.45	0.40			
LS Mean (Puberty)		6.94 ^b^	0.15	10.00 ^a^	0.07					
M11	Male	3.78	0.22	3.99	0.07	3.89 ^A^	0.11	0.010	0.023	0.495
	Female	3.35	0.07	3.73	0.08	3.54 ^B^	0.07			
LS Mean (Puberty)		3.56 ^b^	0.12	3.86 ^a^	0.06					
M12	Male	3.71	0.23	4.09	0.08	3.90 ^A^	0.13	0.022	0.015	0.777
	Female	3.43	0.06	3.73	0.07	3.58 ^B^	0.06			
LS Mean (Puberty)		3.57 ^b^	0.12	3.91 ^a^	0.07					

^a, b^Different letters on the same line indicate a statistically significant difference (*p* < 0.05)

^A, B^Different letters on the same column indicate a statistically significant difference (*P* < 0.05)

Highly significant differences were identified for M1–M10 (*p* < 0.001), while M11 (*p* = 0.023) and M12 (*p* = 0.015) also demonstrated statistically significant pubertal effects. Across the dataset, postpubertal measurements were consistently greater than prepubertal values, indicating substantial morphometric enlargement associated with pubertal maturation.

In addition to the effects of pubertal status, gender-related differences were observed in the majority of the examined parameters. Significant gender effects were detected for M1, M2, M3, M5, M6, M7, M8, M11, and M12 (*p* < 0.05). In contrast, M4, M9, and M10 did not exhibit statistically significant gender-related variation. Comparisons based on least squares means further demonstrated that certain parameters exhibited higher values in males (e.g., M1), whereas others were greater in females (e.g., M2 and M3). These findings may indicate the presence of sexual dimorphism in several morphometric measurements.

A significant interaction between gender and pubertal status (gender × puberty) was detected for M1, M2, M4, M7, and M9, indicating that the magnitude of pubertal changes differed between males and females. Notably, the transition from the prepubertal to the postpubertal stage was more pronounced in females for specific parameters, highlighting the presence of sex-dependent growth patterns during pubertal development.

Collectively, these findings suggest that pubertal maturation may play a major role in morphometric variation, while gender may contribute to additional anatomical differences in several parameters.

## Discussion

The present study examined the effects of gender and pubertal status on 12 pelvic morphometric parameters derived from 3D reconstructed Wistar rat pelves. The main finding was that pubertal status was the most consistent source of variation, as all measured parameters were significantly greater in postpubertal animals. In addition, several parameters showed significant sex-related differences, and some exhibited a significant gender x puberty interaction. The principal contribution of the present study is therefore not simply the confirmation that the pelvis enlarges with maturation, but the demonstration that pubertal status, gender, and their interaction can be evaluated simultaneously across multiple pelvic dimensions using a 3D morphometric framework. This provides a more integrated description of developmental pelvic variation than earlier studies that focused on either strain differences, single-gender samples, or 2D radiographic observations.

The strong effect of pubertal status observed in the present study is concordant with previous reports showing accelerated skeletal growth during maturation [35–37]. Hughes and Tanner [32], in a longitudinal radiographic study, showed that rat pelvic growth changes markedly around the pubertal period and that growth velocity differs across pelvic components. Our results agree with their general conclusion that puberty is a major determinant of pelvic development. However, the present findings extend that work in two ways. First, we evaluated males and females together within the same analytical design, allowing direct comparison of gender-related and pubertal effects. Second, measurements were obtained from reconstructed 3D models rather than serial radiographs, which permits more detailed characterization of pelvic dimensions and reduces the limitations inherent in projection-based 2D assessment. Thus, the current study supports earlier developmental observations while adding a cross-sectional 3D perspective on how multiple pelvic parameters vary by both gender and pubertal stage.

The gender-related differences identified in several parameters are also broadly consistent with the literature on skeletal sexual dimorphism [4, 13, 14, 38, 39]. However, the present results suggest that gender differences are not uniformly expressed across the pelvis. Some parameters showed clear gender-related effects, whereas others were influenced primarily by pubertal status. This uneven distribution of dimorphism is an important point, because it indicates that pelvic development should not be interpreted as a single homogeneous process. Rather, different pelvic dimensions appear to respond differently to maturation and gender-related biological influences. In this respect, the present study adds more specific information than earlier work that described males as simply having larger pelves overall.

The significant gender x puberty interaction observed for several measurements is one of the more informative findings of the study. This result indicates that male-female differences are not constant across developmental stages and that the effect of puberty depends, at least for some parameters, on gender. This interpretation is concordant with Berdnikovs et al. [4], who showed that male and female pelvic developmental trajectories diverge around the onset of puberty. At the same time, our findings are not fully identical to theirs. Berdnikovs et al. emphasized that shape dimorphism emerges earlier than size dimorphism, whereas the present study was based on absolute linear measurements and therefore primarily captures dimensional change rather than shape variation independent of size. Accordingly, the agreement between the two studies lies in the timing and gender dependence of pelvic divergence, while the apparent difference lies mainly in the type of morphometric information analyzed. This methodological distinction is critical and helps explain why direct one-to-one comparison between studies should be made cautiously.

Another important comparison concerns previous work on rat pelvic morphology that focused on inter-strain variation. Üstündağ and Yılmaz [33] reported significant differences in pelvic inlet-related measurements among rat strains, but their study was restricted to adult males. Our findings are not contradictory to that report; rather, the two studies address different sources of variation. Their results indicate that genetic background contributes to pelvic morphology, whereas the present study shows that developmental stage and gender are also major determinants. Methodologically, the inclusion of both genders and two pubertal stages in the current design allows separation of biological influences that could not be evaluated in an adult male-only sample. This distinction strengthens the novelty of the present work, because it shows that pelvic morphometry in rats cannot be fully understood from strain comparisons alone.

An additional observation was the modest right–left difference identified between M9 and M10 measurements in postpubertal males. Although the magnitude of this asymmetry was relatively small, it may reflect natural biological variation associated with skeletal growth and remodeling. Functional asymmetry resulting from preferential limb use has been reported in mammalian skeletal structures and may contribute to minor morphometric differences. However, because digital segmentation and landmark identification procedures can also introduce small measurement variations, a methodological contribution cannot be completely excluded. Future studies involving larger sample sizes, bilateral symmetry analyses, and longitudinal designs would help determine whether these differences represent true biological asymmetry or measurement-related variation.

It should also be noted that considerable differences in body weight existed among the experimental groups. Postpubertal males and females were substantially heavier than prepubertal males and females. Consequently, part of the observed morphometric variation may reflect overall somatic growth rather than exclusively changes in pelvic morphology. Because the present analyses were based on absolute morphometric measurements and did not include body-size-adjusted statistical models, the findings should be interpreted as representing the combined effects of growth, maturation, and sexual dimorphism. Future studies incorporating allometric scaling or body-weight-adjusted analyses would help distinguish shape-related changes from those attributable to overall body size.

Several limitations should be considered when interpreting the present findings. First, although standardized digital measurement procedures and repeated observer assessments were employed, minor measurement variability cannot be entirely excluded. Second, the study focused exclusively on morphometric parameters and did not include complementary histological, biomechanical, or functional analyses that could provide further insight into the biological mechanisms underlying pelvic development. Third, the cross-sectional design allows comparison between developmental stages but does not permit direct evaluation of individual growth trajectories. Longitudinal studies following animals throughout maturation would provide more detailed information regarding the timing and progression of pelvic development. In addition, no allometric correction or geometric morphometric analysis was performed. Because developmental groups differed substantially in body size, some of the observed variation may reflect differences in overall growth rather than shape alone. Finally, although an a priori power analysis indicated that the sample size was sufficient to detect the expected effects, larger cohorts may improve the detection of subtle interaction effects and increase the generalizability of the findings.

## Conclusion

The findings of the present study suggest that pubertal development is associated with measurable changes in pelvic morphometric parameters in Wistar rats. Several parameters also exhibited sex-related differences, indicating the potential presence of sexual dimorphism during skeletal maturation. Furthermore, the observed gender × puberty interaction effects may reflect differences in growth trajectories between males and females during development. Although additional studies with larger sample sizes and longitudinal designs are needed, the present results may provide useful baseline morphometric data for future investigations of pelvic development, skeletal maturation, and sexual dimorphism in experimental animal models.

## Data Availability

The datasets generated and/or analysed during the current study are available from the corresponding author on reasonable request.

## References

[1] Islam MM, Farag E, Mahmoudi A, Hassan MM, Atta M, Mostafavi E, Alnager IA, Farrag HA, Eljack GEA, Bansal D, Haroun M, Abdeen R, Al-Romaihi H, Al-Zeyara AA, Almalki SA, Mkhize-Kwitshana Z. Morphometric Study of Mus musculus, Rattus norvegicus, and Rattus rattus in Qatar. Anim (Basel). 2021;11(8):2162. 10.3390/ani11082162. PMC8388387 34438620

[2] Tan Y, Tan D, Anatomy. Physiology, and Husbandry of Laboratory Animals. In: Liu E, Fan J, editors. Fundamentals of Laboratory Animal Science. 1st ed. Boca Raton: CRC; 2017. Chapter 4. 10.1201/9781315368993.

[3] Sengupta P. The Laboratory Rat: Relating Its Age With Human’s. Int J Prev Med. 2013;4(6):624–30. PMC3733029 23930179

[4] Berdnikovs S, Bernstein M, Metzler A, German RZ. Pelvic growth: Ontogeny of size and shape sexual dimorphism in rat pelves. J Morphol. 2007;268:12–22. 10.1002/jmor.10476. 17154287

[5] Tague RG. Pelvic sexual dimorphism among species monomorphic in body size: relationship to relative newborn body mass. J Mammal. 2016;97(2):503–17. 10.1093/jmammal/gyv195. PMC6169469 30302031

[6] Matysiak A, Malecha AW, Jakubowski H, Sadowska ET, Koteja P, Tryjanowski P. Sexual dimorphism, asymmetry, and the effect of reproduction on pelvis bone in the bank vole, *Myodes glareolus*. Mamm Res. 2017;62:297–306. 10.1007/s13364-017-0317-1.

[7] Buck LT, Katz DC, Ackermann RR, Hlusko LJ, Kanthaswamy S, Weaver TD. Effects of hybridization on pelvic morphology: A macaque model. J Hum Evol. 2021;159:103049. 10.1016/j.jhevol.2021.103049. 34455262

[8] Cox SL. A geometric morphometric assessment of shape variation in adult pelvic morphology. Am J Phys Anthropol. 2021;176(4):652–71. 10.1002/ajpa.24399. 34528241

[9] Fischer B, Grunstra NDS, Zaffarini E, Mitteroecker P. Sex differences in the pelvis did not evolve de novo in modern humans. Nat Ecol Evol. 2021;5(5):625–30. 10.1038/s41559-021-01425-z. PMC7617076 33767411

[10] Parent AS, Teilmann G, Juul A, Skakkebaek NE, Toppari J, Bourguignon JP. The timing of normal puberty and the age limits of sexual precocity: variations around the world, secular trends, and changes after migration. Endocr Rev. 2003;24(5):668–93. 10.1210/er.2002-0019. 14570750

[11] Styne DM, Grumbach MM, Puberty. Ontogeny, Neuroendocrinology, Physiology, and Disorders. In: Melmed S, Polonsky KS, Larsen PR, Kronenberg HM, editors. Williams Textbook of Endocrinology. 12th ed. Philadelphia: Saunders; 2011. pp. 1054–201. 10.1016/B978-1-4377-0324-5.00025-0.

[12] Uesugi Y, Taguchi O, Noumura T, Iguchi T. Effects of sexsteroids on the development of sexual dimorphism in mouse innominate bone. Anat Rec. 1992;234(4):541–8. 10.1002/ar.1092340409. 1456456

[13] Seeman E. Clinical review 137: Sexual dimorphism in skeletal size, density, and strength. J Clin Endocrinol Metab. 2001;86(10):4576–84. 10.1210/jcem.86.10.7960. 11600506

[14] Riggs BL, Khosla S, Melton LJ. Sex steroids and the construction and conservation of the adult skeleton. Endocr Rev. 2002;23(3):279–302. 10.1210/edrv.23.3.0465. 12050121

[15] Tague RG. Variation in pelvic size between males and females in nonhuman anthropoids. Am J Phys Anthropol. 1995;97(3):213–33. 10.1002/ajpa.1330970302. 7573375

[16] Freitas EP, Noritomi PY, Silva JVL. Use of rapid prototyping and 3D reconstruction in veterinary medicine. In: Hoque ME, editor. Advanced Applications of Rapid Prototyping Technology in Modern Engineering. InTech; 2011. Chapter 7. 10.5772/23303.

[17] Wilhite R, Wölfel I. 3D Printing for veterinary anatomy: An overview. Anat Histol Embryol. 2019;48(6):609–20. 10.1111/ahe.12502. 31702827

[18] Jamali AA, Deuel C, Perreira A, Salgado CJ, Hunter JC, Strong EB. Linear and angular measurements of computer-generated models: are they accurate, valid, and reliable? Comput Aided Surg. 2007;12(5):278–85. 10.3109/10929080701680265. 17957535

[19] Stull KE, Tise ML, Ali Z, Fowler DR. Accuracy and reliability of measurements obtained from computed tomography 3D volume rendered images. Forensic Sci Int. 2014;238:133–40. 10.1016/j.forsciint.2014.03.005. 24713466

[20] Ogden KM, Aslan C, Ordway N, Diallo D, Tillapaugh-Fay G, Soman P. Factors Affecting Dimensional Accuracy of 3-D Printed Anatomical Structures Derived from CT Data. J Digit Imaging. 2015;28(6):654–63. 10.1007/s10278-015-9803-7. PMC4636725 25982877

[21] Özkadif S, Eken E. Craniometric Measurements of New Zealand Rabbits Skull from Three-Dimensional Reconstruction Images. Ajavs. 2016;2(1):9–14. 10.20431/2455-2518.0201002.

[22] Özkadif S, Eken E, Kalaycı I. A three-dimensional reconstructive study of pelvic cavity in the New Zealand rabbit (*Oryctolagus cuniculus*). ScientificWorldJournal. 2014;2014:489854. 10.1155/2014/489854. PMC4212587 25379534

[23] Selçuk ML, Turgut N. Pelvis Anatomy and Morphometric Analysis in New Zealand Rabbits. Veterinary Sci Practices. 2024;19(2):104–11. 10.17094/vetsci.1447528.

[24] Özkadif S, Eken E, Beşoluk K, Dayan MO. Three-dimensional reconstruction of New Zealand rabbit antebrachium by multidetector computed tomography. Iran J Vet Res. 2015;16(2):205–9. PMC4827687 27175177

[25] Özkadif S, Eken E. Three-dimensional reconstruction of multidetector computed tomography images of paranasal sinuses of New Zealand rabbits. Turk J Vet Anim Sci. 2013;37(6):675–81. 10.3906/vet-1301-53.

[26] Remzi OA, Caner B, Okan E, Ufuk K, Nuriye ÖK. Accuracy and reliability of measurements obtained from 3-dimensional rabbit mandible model: a micro-computed tomography study. Acta Vet-Beogr. 2019;69(2):192–200. 10.2478/acve-2019-0015.

[27] Akgün RO, Orhan İÖ, Ekim O. Three-dimensional bone modeling of forelimb joints in New Zealand Rabbit: A Micro-Computed Tomography study. Ankara Univ Vet Fak Derg. 2021;68(4):355–63. 10.33988/auvfd.762615.

[28] Bakici C, Akgun RO, Ekim O, Batur B, Bakici M, Ozen D, Soydal C. Three dimensional modeling and quantitative analysis of long bone parameters of rabbit using micro-computed tomography. Iran J Vet Res. 2021;22(2):140–5. 10.22099/ijvr.2021.39092.5688. PMC8294823 34306112

[29] Dayan MO, Beşoluk K, Eken E, Aydoğdu S, Turgut N. Three-dimensional modelling of the femur and humerus in adult male guinea pigs (guinea pig) with computed tomography and some biometric measurement values. Folia Morphol (Warsz). 2019;78(3):588–94. 10.5603/FM.a2019.0002. 30644082

[30] Baygeldi SB, Yılmaz Y, Güzel BC, Kanmaz YA, Karan M, Hark BD, Yılmaz S. Analysis and 3D imaging of morphometric- mineral structure changes in skulls of male and female guinea pigs during and after developmental period. Int J Morphol. 2024;42(2):530–7. 10.4067/S0717-95022024000200530.

[31] Counter WB, Wang IQ, Farncombe TH, Labiris NR. Airway and pulmonary vascular measurements using contrast-enhanced micro-CT in rodents. Am J Physiol Lung Cell Mol Physiol. 2013;304(12):L831–43. 10.1152/ajplung.00281.2012. 23564512

[32] Hughes PC, Tanner JM. A radiographic study of the growth of the rat pelvis. J Anat. 1973;114(Pt 3):439–48. PMC1271455 4351911

[33] Üstündağ Y, Yılmaz O. Comparative anatomy of the pelvic cavity of rat strains in a translational aspect. Harran Univ Vet Fak Derg. 2025;14(1):31–8. 10.31196/huvfd.1621777.

[34] International Committee on Veterinary Gross Anatomical Nomenclature. Nomina Anatomica Veterinaria. 6th ed. World Association of Veterinary Anatomists (WAVA); 2017.

[35] Karlberg J. On the modelling of human growth. Stat Med. 1987;6(2):185–92. 10.1002/sim.4780060210. 3589247

[36] Tanner JM. Fetus into Man: Physical Growth from Conception to Maturity. Revised and Enlarged Ed. Cambridge: Harvard University Press; 1990. pp. 58–84. 10.1002/ajhb.1310030224.

[37] Bogin B. Patterns of Human Growth. 2nd ed. Cambridge: Cambridge University Press; 1999. pp. 54–149.

[38] Fairbairn DJ, Blanckenhorn WU, Székely T, Sex. Size and Gender Roles: Evolutionary Studies of Sexual Size Dimorphism. 1st ed. Oxford: Oxford University Press; 2007.

[39] Gilsanz V, Ratib O. Hand bone age: a digital atlas of skeletal maturity. 2nd ed. Berlin Heidelberg: Springer; 2012. p. 3–18.

